# The utility of initial procalcitonin and procalcitonin clearance for prediction of bacterial infection and outcome in critically ill patients with autoimmune diseases: a prospective observational study

**DOI:** 10.1186/s12871-015-0122-9

**Published:** 2015-10-07

**Authors:** Yan Shi, Jin-min Peng, Xiao-yun Hu, Yao Wang

**Affiliations:** 1 Department of general intensive care unit, Peking Union Medical College Hospital, Peking Union Medical College, Chinese Academy of Medical Sciences, Beijing, China; 2 Department of medical intensive care unit, Peking Union Medical College Hospital, Peking Union Medical College, Chinese Academy of Medical Sciences, Beijing, China; 3 Department of Clinical Laboratory, Peking Union Medical College Hospital, Peking Union Medical College, Chinese Academy of Medical Sciences, Beijing, China

**Keywords:** Procalcitonin, Procalcitonin clearance, Autoimmune diseases, Critically ill patients, Bacterial infection, Outcome

## Abstract

**Background:**

The diagnostic value of procalcitonin (PCT) for patients with autoimmune diseases (AID) remains controversial and few studies focused on ICU patients. We sought to determine its diagnostic and prognostic values in this clowd.

**Methods:**

A prospective observational study was conducted in AID patients admitted to the ICU. Serum PCT levels were measured on ICU admission and subsequently at days 1, 3, 5 and 7, and peak PCT levels within 24 h (PCT_peak_) were analyzed the utility for bacterial infection. The relationship of PCT_peak_ and SOFA score and severity of sepsis was performed correlation analysis. The change of PCT over time reflected as PCT clearance was compared to ICU 28-day mortality.

**Results:**

One hundred twelve patients were divided into bacterial infection group (group I, *n* = 54) and nonbacterial condition group (group II, *n* = 58). The median PCTpeak (range, μg/L) was higher in the group I than that in the group II (1.95 [0.38–37.56] vs. 0.64 [0.05–7.83], *p* = 0.002). PCTpeak had the best single predictor of bacterial infection (area under the curve [AUC], 0.902, *p* < 0.001) with a sensitivity of 79.6 % and a specificity of 89.6 % at the threshold of 0.94 μg/L. PCTpeak was also positive correlation with severity of sepsis (*r* = 0.731, *p* = 0.002), but its correlation with SOFA score was only found in subjects with bacterial infection (*r* = 0.798, *p* < 0.001). Importantly, the 5-day PCT clearance (PCTc-d5), rather than absolute PCT values, could earlier discriminate survivors (*n* = 73) from nonsurvivors (*n* = 39) (68.8 ± 9.8 vs. 21.8 ± 17.5 %, *p* < 0.001, respectively). PCTc-d5 < 50 % was an independent predictor of mortality (odds ratio 5.1, 95 % confidence interval 3.5 to 7.5; *p* = 0.001).

**Conclusions:**

In critically ill patients with AID, elevated PCT levels are valuable for bacterial infection and are significantly positive correlation with the septic severity. Five-day PCT clearance may provide independent prognostic information. Larger, prospective trials are warranted to confirm the benefit.

**Electronic supplementary material:**

The online version of this article (doi:10.1186/s12871-015-0122-9) contains supplementary material, which is available to authorized users.

## Background

Patients with autoimmune disease (AID) are more susceptible to systemic bacterial infection with worse outcome [1, 2]. Due to their compromised immune statuses, clinical, laboratory and molecular signs for infection are often subtle and nonspecific. Furthermore, it has been shown that inflammatory signs can be masked by immunosuppressants or corticosteroid and render C-reactive protein (CRP) inconsistently reliable as a biomarker for bacterial infection in this setting [3, 4]. So, there is an urgent need for a reliable biomarker for the early diagnosis.

Over the last decade, Procalcitonin (PCT), a 116-amino acid polypeptide and a precursor of calcitonin, has become increasingly popular as a marker for bacterial infection. It is well known that PCT is positively correlated with bacterial infection and severity of infection, but also is not attenuated by steroids and has a more helpful than CRP [5, 6]. Unfortunately, PCT has not been extensively studied in patients with AID. The few currently available studies provided conflicting results [7–10]. Especially for critically ill patients, PCT is still some debate because it may increase nonspecifically in a variety of noninfectious conditions (e.g., systemic inflammation, cardiogenic shock, organ dysfunction, and tissue trauma) [5, 6]. Therefore, Little is known about the variation of initial PCT levels and its clearance in ICU patients with AID.

We described our preliminary experience in published letter [11]. In this prospective observational study, we sought to further define the potential role of PCT for bacterial infection in this crowd. We measured PCT on admission (day 0) and within 6 to 24 h after admission (day 1), and the peak PCT concentration (PCT_peak_) was used to assess the diagnostic utility of bacterial infection. In order to distinguish organ-dysfunction related induction and infection-related PCT response, we also studied PCT concentrations in patients with or without bacterial infection at a similar severity of organ dysfunction. Furthermore, we performed PCT determinations every other day through 1 week and hypothesised that early change of PCT over time, or defined as PCT clearance, is associated with ICU 28-day mortality.

## Methods

### Setting and participants

All AID patients (18 years or older) who were admitted to medical ICU of our hospital between January 1, 2011 and December 31, 2013 were consecutively screened. Patients with small cell lung cancer, medullary thyroid carcinoma, trauma or surgery which are served as the common non-infectious causes of PCT elevations were excluded. In addition, the following conditions will be excluded from the final analysis for accurate classification: if they had suspected infection but no pathogen could be identified; if they had uncertain diagnosis as well as infection with disease flare at the same time; if they had incomplete data or ICU length of stay (LOS) of less than 7 days.

This study was approved by the ethics committee of Academy of Medical Sciences & Peking Union Medical College Hospital (Approval Number: S-112) who waived the need for informed consent in consideration of the anonymous collection of data and observational nature of the study.

### Data collection

The following data were recorded upon ICU admission: demographic characteristics, comorbidities, the type of AID and the median time of diagnosis of AID, the median steroid dose and use of other immunosuppressants in the 3 months before admission, the use of high-dose steroid before ICU admission, prior hospitalization, reasons for ICU admission, suspected of having a bacterial infection and severity of organ dysfunction expressed by the Sepsis-related Organ Failure Assessment (SOFA) score [12]. During follow up, organ support (mechanical ventilation, renal replacement therapy, and use of vasopressors), finally confirmed infection, sources of infection, microbiological findings and septic condition (that is, sepsis, severe sepsis or septic shock) according to American College of Chest Physicians/Society of Critical Care Medicine criteria [13], appropriate treatment of the primary cause, ICU LOS and crude ICU 28 day-mortality were also recorded.

### Treatments and interventions

Patients were examined and treated according to the practice of the institution. Empirical antimicrobial therapy for suspected infection was administrated, which was modified according to ongoing microbiological and laboratory results. Routine cultures of blood, urine, and of samples from trachea and suspected sites and serologic test, or nucleic acid determination were taken for diagnosis. The microbiological diagnostic workup included a search for *Legionella pneumophila*, *Chlamydia pneumoniae*, *Mycoplasma pneumoniae*, and respiratory viruses (*ie, herpes simplex virus, cytomegalovirus, respiratory syncytial virus, and adenovirus*) by polymerase chain reaction, culture, or immunofluorescence. Appropriate stains and cultures for bacteria, mycobacteria, fungi, and *Pneumocystis jiroveci* were performed. Chest radiographs were performed in all cases, and CT scans were obtained as indicated by the treating physicians.

### Laboratory assay and PCT measurements

All assays were performed according to the manufacturers’ instructions and standard microbiology guidelines [14]. Lower respiratory samples were obtained by tracheal aspiration, bronchoalveolar lavage or sputum specimen. Only high-quality specimens (<10 epithelial cells and > 25 white blood cells per low-power field) were cultured, and more than 3+ of bacterial growths using a semiquantitative culture method were considered positive.

PCT and laboratory tests (blood routine, liver and kidney function test) were prospectively measured on ICU admission (day 0) and subsequently at days 1, 3, 5 and 7. PCT measured using a commercially available immunoluminometric assay system (Brahms Diagnostica, Hennigsdorf bei Berlin, Germany) following the manufacturer’s protocols. The lower detection limit is 0.05 μg/L.

### Grouped and definition

The final determination of the patient’s diagnosis was done retrospectively during a meeting of the ICU and rheumatology physicians after patient discharge based on results of microbiological cultures, radiographs and serologic test.

The diagnosis categorized as either having a proven bacterial infection (including co-infections of bacterial, fungal and /or viral) or having a nonbacterial condition, which was further divided into nonbacterial infection (i.e., fungus or virus) and noninfectious conditions (i.e., disease flare or others). The bacterial infection was defined as positive pathogen results from infection site or blood, and with objective signs and symptoms and radiographs according to International Sepsis Forum Consensus Conference recommendations [15]. The diagnosis of fungal infection was only included in the classification of proven and probable based on the definitions of the European Organization for the Research and Treatment of Cancer-Mycoses Study Group (EORTC-MSG) [16]. The diagnosis of viral infection was established in a patient with positive serum samples or positive polymerase chain reaction tests from cerebrospinal fluid or blood [17]. The diagnosis of a disease flare was made when there was a significant increase in the activity score compared to the previous condition which was not caused by any other conditions (such as infection or drug). The disease activity index was calculated based on the disease Activity Score or standard [18–24].

### Statistical methods

All data are expressed as the mean (± SD) or median (range) as appropriate. The *χ*2 test or Fisher’s exact test was used to compare categorical variables. The Student’s *t*-test or Mann–Whitney *U* test was used to compare continuous variables. Univariate and multivariate analysis were used to identify factors associated with mortality. Variables yielding *p* values < 0.2 by univariate analysis, or those considered clinically relevant despite *p*-values, were entered in the multivariate analysis. A *p*-value of < 0.05 was considered statistically significant. Data were analyzed using SPSS statistical software (version 17.0; SPSS Inc., Chicago, IL, USA).

PCT_peak_ was considered for analyses of diagnosis accuracy and expressed as the area under the receiver operating characteristic curve (AUC). Among PCT_peak_, SOFA score and septic conditions, pearson's correlation and the regression formula were calculated (*y* = *a* + *bx*). PCT clearance (PCTc) was defined as percentage of delta PCT (ΔPCT) concentration which is PCT_peak_ minus PCT measured in the subsequent over PCT_peak_. The formula is as follows: PCTc-dx = (PCT_peak_ – PCT_dX_) / PCT_peak_. The values are negative with increasing concentrations and positive with decreasing concentrations.

## Results

### Patients’ characteristics and grouping

**Fig. 1:**
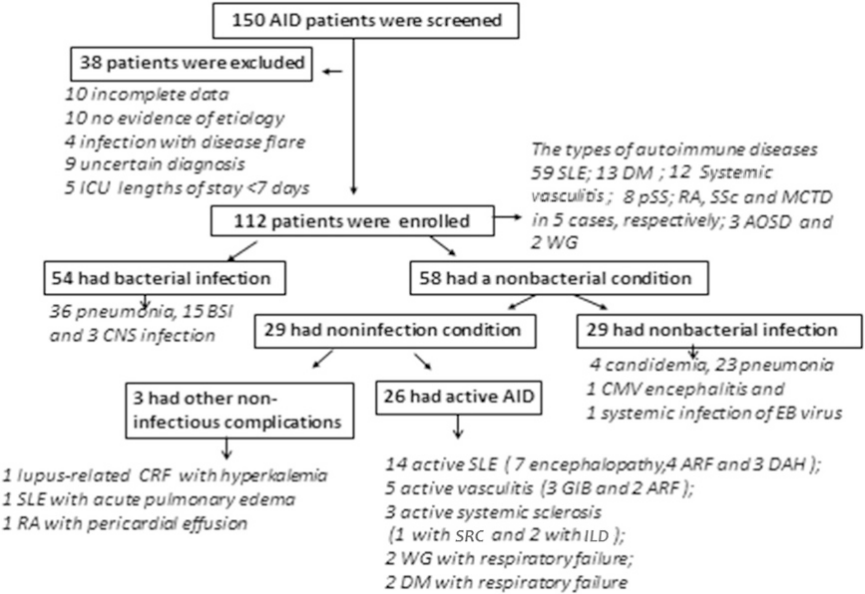
Study design showing patient allocation and diagnosis. *SLE: systemic lupus erythematosus; DM: dermatomyositis; pSS: primary Sjogren’s syndrome; RA: rheumatoid arthritis; SSc: systemic sclerosis; MCTD: mixed connective tissue diseas; AOSD: adult-onset Still’s disease; WG: Wegener’s granulomatosis; BSI: bloodstream infection; CNS: central nervous system; CRF: chronic renal failule; ARF: acute renal failure; DAH: diffuse alveolar hemorrhage; GIB: gastrointestinal bleeding; SRC: Sclerode rmarenal crisis; ILD: interstitial lung disease*

One hundred twelve patients (44 males and 68 females) selected among 150 AID patients were included. The types of AID and the reasons for exclusion are listed in Fig. 1. The patients’ age was 53.0 ± 17.1 years and median time of diagnosis of AID was 10.8 months (range, 2 weeks to 13 years). SOFA score on ICU admission was 6.9 ± 3.8 points. 99 patients need for mechanical ventilation, 18 need for renal replacement therapy and 55 need for vasopressor, respectively. ICU LOS and 28-day crude ICU mortality were 12.5 (7 to 33) days and 34.8 % (39 of 112), respectively. The patients’ characteristics are summarized in Table 1.

**Table 1:** Patients’ characteristics and outcome

	Bacterial infection	Nonbacterial condition	Survivors	Non-survivors
	*n* = 54	*n* = 58	*n* = 73	n = 39
Demographic characteristics				
Age, mean ± SD, yr.	52.5 ± 13.1	51.8 ± 15.8	53.1 ± 11.7	52.3 ± 17.6
Female, *n* (%)	33 (61.1 %)	38 (65.5 %)	47 (64.4 %)	24 (61.5 %)
Type of AID, *n* (%)				
SLE	30 (55.6 %)	29 (50 %)	44 (60.3 %)	15 (38.5 %)^b^
Systemic vasculitis	5 (9.3 %)	7 (12.1 %)	7 (9.6 %)	5 (12.8 %)
Dermatomyositis	3 (5.6 %)	10 (17.2 %)^a^	4 (5.5 %)	9 (23.1 %)^a^
pSS	4 (7.4 %)	4 (6.9 %)	6 (8.2 %)	2 (5.1 %)
RA	3 (5.6 %)	2 (3.4 %)	3 (4.1 %)	2 (5.1 %)
SSc	3 (5.6 %)	2 (3.4 %)	3 (4.1 %)	2 (5.1 %)
MCTD	3 (5.6 %)	2 (3.4 %)	3 (4.1 %)	2 (5.1 %)
Others	3 (5.6 %)	2 (3.4 %)	3 (4.1 %)	2 (5.1 %)
Comorbid conditions, *n* (%)				
Heart function grade > 3 (NYHA)	4 (7,4 %)	6 (10.3 %)	3 (4.1 %)	7 (17.9 %)^a^
Chronic renal insufficiency (CKD stage > 3)	5 (9.3 %)	6 (10.3 %)	4 (5.5 %)	7 (17.9 %)^a^
Prior hospitalization, days, median (range)	3.3 (1–38)	3.0 (1–33)	3.2 (1–35)	2.9 (1–38)
The treatment before ICU admission				
The median equivalent prednisone dose, mg/d	43.2 ± 22.4	41.4 ± 25.2	40.2 ± 25.7	44.4 ± 21.5
Immunosuppressants, *n* (%)	11 (20.4 %)	13 (22.4 %)	18 (24.7 %)	6 (15.4 %)^b^
High dose steroid, *n* (%)	6 (19.4 %)	9 (31.0 %)^b^	5 (6.8 %)	10 (25.6 %)^a^
Antibiotic therapy, *n* (%)	44 (81.4 %)	50 (86.2 %)	61 (83.6 %)	33 (84.6 %)
Reasons for ICU admission, *n* (%)				
Acute respiratory failure	24 (44.4 %)	24 (41.4 %)	26 (35.6 %)	22 (56.4 %)^b^
Acute kidney failure	1 (1.9 %)	5 (8.6 %)^a^	5 (6.8 %)	1 (2.6 %)
Conscious disturbance	3 (5.6 %)	8 (13.8 %)^a^	4 (5.5 %)	7 (17.9 %)^a^
Hypopressure of any reasons	10 (14.8 %)	8 (13.8 %)	14 (19.2 %)	4 (10.3 %)^b^
The coexistence of hypotension and respiratory failure	16 (29.6 %)	13 (22.4 %)	16 (21.9 %)	13 (33.3 %)^b^
Characteristics on admission				
Body temperature, °C	38.2 ± 0.5	38.3 ± 0.7	38.3 ± 0.6	38.3 ± 0.3
Leucocytes, cells/mm^3^	10,300 ± 7200	10,100 ± 5600	10,100 ± 6400	10,200 ± 7600
Granulocyte, %	88.5 ± 18.9	88.1 ± 21.5	88.2 ± 15.1	87.9 ± 13.6
Neutropenia, *n* (%)	4 (7.4 %)	4 (6.9 %)	5 (6.8 %)	3 (7.7 %)
Serum creatinine, μmol/l	106.7 ± 28.1	108.2 ± 35.5	102.5 ± 35.5	110.5 ± 23.7
SOFA score, points	6.8 ± 3.1	6.2 ± 3.5	5.4 ± 2.2	8.9 ± 2.1^a^
Organ support, *n* (%)				
mechanical ventilation	48 (88.9 %)	51 (87.9 %)	60 (82.2 %)	39 (100 %)^b^
renal replacement therapy	8 (14.8 %)	10 (17.2 %)	7 (11.0 %)	11 (28.2 %)^a^
vasopressor	26 (48.1 %)	29 (50.0 %)	30 (41.1 %)	25 (64.1 %)^a^
ICU new acquired severe sepsis, *n* (%)	18 (33.3 %)	23 (39.7 %)	21 (28.8 %)	20 (51,3 %)^a^
Duration of shock, days, median (range)	6.5 (3–15)	6.9 (2–14)	3.8 (2–10)	7.5 (7–15)^a^
ICU stay, days, median (range)	13.1 (8–31)	13.6 (7–33)	12.5 (8–29)	13.9 (7–33)
Appropriate treatment of the primary cause, *n* (%)	28 (51.8 %)	26 (44.8 %)	42 (57.5 %)	12 (30.8 %)^a^
28-day ICU mortality, *n* (%)	18 (33.3 %)	21 (36.2 %)	-	-

*AID* autoimmune diseases; *SLE* systemic lupus erythematosus; *pSS* primary Sjogren’s syndrome

*RA* rheumatoid arthritis; *SSc* systemic sclerosis; *MCTD* mixed connective tissue disease

^a^*P* < 0.05 versus the control group; ^b^*P* < 0.20 versus the control group

Of the 112 patients, 54 (48.2 %) had a bacterial infection (group I) and 58 had a nonbacterial condition (group II). In the latter, nonbacterial infection and noninfectious condition occurred in 29, respectively. In the group I, the sourses of infection were as follows: 15 bloodstream infection, 36 pulmonary and 3 central nervous system infection. The main organisms were gram negative bacilli (43/54, 79.6 %) and co-infection accounted for 27.8 % (15/54). In the group of nonbacterial infection, the diagnosis was 4 candidemia, 23 nonbacterial pneumonia, one cytomegalovirus (CMV) encephalitis and one systemic infection of EB virus (All etiological data are presented in Additional file 1). Moreover, the compositions of noninfectious conditions were 26 cases of disease flares and three cases of other noninfectious complications (Fig. 1). In patients with disease activities, the manifestations are as follows: 14 active SLE patients (SLEDAI score 13.8 ± 5.4 points); five of active *microscopic polyangiitis* (BVAS 16.5 ± 5.6 points); six cases with respiratory failure (including 2 of *systemic sclerosis*, *Wegener’s granulomatosis* and *dermatomyositis,* respectively) due to connective tissue disease related interstitial lung disease and one with scleroderma renal crisis.

### PCT for initial diagnosis

Of all patients, the median PCT_peak_ (range) was 1.88 (0.05–37.56) μg/L and delayed PCT_peak_ occurred in 28 patients and were as follows: 10 of new onset bacteremia, 9 of bacterial pneumonia, 6 of active AID (SLE related diffuse alveolar hemorrhage, vasculitis related *gastrointestinal bleeding* and WG in two cases, respectively), 2 of candidemia and one of SLE with acute pulmonary edema. The median PCT_peak_ (range, μg/L) was higher in the group I than that in the group II (1.95 [0.38–37.56] vs. 0.64 [0.05–7.83], *p* = 0.002), but similar in the subgroup of nonbacterial infection and noinfectious condition (0.61 [0.08–7.83] vs. 0.67 [0.05–7.78], *p* = 0.917), respectively.

**Fig. 2:**
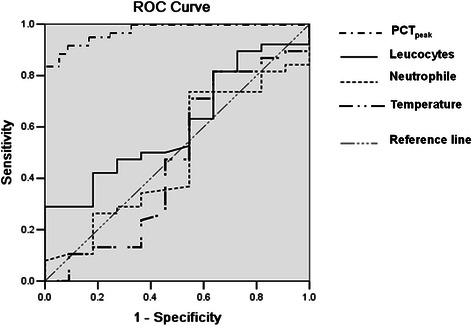
Receiver operating curve (ROC) analysis on discrimination of bacterial infection. *Peak PCT levels within 24 h (PCT*_*peak*_*) had the better performance for bacterial infection with an AUC of 0.902 and had a sensitivity of 79.6 %, specificity of 89.6 % at the threshold of 0.94 μg/L*

Based on clinical evaluations and PCT guide (>0.5 μg/L), 92 cases and 67 cases were diagnosed as suspected of having a bacterial infection on admission, respectively. PCT_peak_ had the better performance for bacterial infection with an AUC of 0.902 (95 % CI 0.846–0.959; *p* < 0.001) as compared to temperature (AUC = 0.472; 95 % CI 0.256–0.689; *p* = 0.783), leucocytes (AUC = 0.606, 95 % CI 0.432–0.681; *p* = 0.286), neutrophil percentage (AUC = 0.484, 95 % CI 0.291–0.678; *p* = 0.876) and clinical comprehensive evaluation (AUC = 0.651, 95 % CI 0.491–0.759; *p* = 0.166). The cutoff point of 0.94 μg/L had a sensitivity of 79.6 %, specificity of 89.6 %, positive likelihood ratio of 7.65, negative likelihood ratio of 0.22 and an accuracy of 86.8 % for predicting bacterial infection (Fig. 2).

### PCT and severity of organ dysfunction and septic condition

PCT_peak_ of all patients showed positive correlation with severity of organ dysfunction expressed by the SOFA score (*r* = 0.698, *p* < 0.001) (Table 2), but strongly positive correlation was only found in subjects with bacterial infection (*r* = 0.798, *p* < 0.001), rather than in those without (*r* = 0.205, *p* = 0.791). It was expressed with regression equation (*y* = a + bx) as follows: PCT = − 0.921 + 1.326 SOFA score and PCT = 0.153 + 0.038 SOFA score, respectively. In other words that PCT_peak_ was still higher in patients with bacterial infection than those without at comparable levels of organ dysfunction (Table 3).

**Table 2:** Peak procalcitonin levels (PCT_peak_) at different groups of SOFA score and septic condition

	SOFA	PCT_peak,_ μg/L
Category of SOFA		
1–6 (*n* = 78)	3.5 (2–6)	0.68 (0.05–5.88)
7–12 (*n* = 27)	9.3 (7–11)	3.85 (0.08–9.95)
13–18 (*n* = 5)	14.2 (13–15)	10.58 (4.77–29.90)
19–24 (*n* = 2)	19 (19)	28.74 (19.98–37.56)
Category according to septic condition		
SIRS (*n* = 29)	3.3 (2–13)	0.67 (0.05–7.83)
Sepsis (*n* = 18)	4.1 (3–6)	1.73 (0.05–4.60)*
Severe sepsis (*n* = 24)	6.1 (4–7)**	4.81 (0.08–22.15)**
Septic shock (*n* = 41)	9.8 (6–20)***	15.50 (0.08–37.56)***

Data presented as median values (range). *SIRS,* systemic inflammatory response syndrome

*P < 0.05 versus SIRS, **P < 0.05 versus sepsis, ***P < 0.05 versus severe sepsis

**Table 3:** Procalcitonin (PCT) levels at various severities of organ dysfunction comparing patients with and without bacterial infection

Category of SOFA score (group I/group II)	PCT_peak_		*P* value
	Bacterial infection (group I)	*Nonbacterial condition* (group II)	
SOFA 1–6 (*n* = 32/46)	0.68 (0.59–5.88)	0.14 (0.05–0.33)	0.038
7–12 (*n* = 17/10)	7.8 (3.52–9.95)	0.25 (0.08–3.85)	<0.001
13–18 (*n* = 3/2)	15.5 (13.83–29.90)	6.30 (4.77–7.83)	0.001
19–24 (*n* = 2/0)	28.7 (19.98–37.56)	-	-

Data presented as median values (range)

Othermore, analyzing of PCT_peak_ of 83 patients with sepsis (18 of sepsis, 24 of severe sepsis an 41 of septic shock) demonstrated that PCT_peak_ levels were also positive correlation with the severity of sepsis (*r* = 0.731, *p* = 0.002) and higher PCT_peak_ may progress to severe sepsis or septic shock (Table 2).

### PCT clearance (PCTc) and prognosis

PCT concentrations (μg/L) were similar between survivors and nonsurvivors (include both infectious and non-infectious patients) throughout the 3-day period (PCT_peak_: 1.92 [0.10–37.56] vs. 1.83 [0.10–31.33], *p* = 0.79; PCT_d3_: 1.35 [0.08–57.20] vs. 1.43 [0.08–25.92], *p* = 0.67, survivors vs. nonsurvivors, respectively). Despite PCT_d5_ had a decline tendency in survivors than in nonsurvivors (1.03 [0.08–38.38] vs. 1.47 [0.08–37.73], respectively, *p* = 0.06), PCT_d7_ was found significantly lower (0.53 [0.08–17.1] vs. 2.15 [0.08–110.10], respectively, *p* = 0.005). By comprasion, 5-day PCT clearance (PCTc-d5) earliest showed significant difference between 2 groups (68.8 ± 9.8 vs. 21.8 ± 17.5 %, *p* < 0.001, survivors vs. nonsurvivors, respectively). On the contrary, the trendency of leukocytosis, temperature and granulocyte percentage did not differ between 2 groups (p > 0.05 all). In particular, PCTc-d5 was significantly faster in the survivors of bacterial infection compared with those without (80.8 ± 15.7 vs. 35.8 ± 14.5 %, respectively, *p* < 0.001).

PCT levels at days 1, 3, 5 and 7, PCT clearance and other 17 prognostic variables with a *p*-value of <0.2 by univariate analysis (Table 1) were included in the multivariable logistic regression analysis and demonstrated five independent predictors of 28-day mortality. These included PCTc-d5 < 50 % (odds ratio [OR] 5.1, 95 % confidence interval [CI] 3.5 to 7.5, *P* = 0.01), SOFA > 8.9 score (OR 2.5, 95 % CI 1.4 to 3.6, *P* = 0.02), duration of shock > 7 days (OR 3.6, 95 % CI 1.6 to 7.8, *p =* 0.02), inappropriate therapy of the primary cause (OR = 2.0, 95 % CI 1.1 to 7.6, *p* = 0.03) and ICU new acquired severe sepsis (OR 5.8, 95 % CI 3.2 to 8.7, *p* = 0.01).

## Discussion

Early predicting severe infection remains a major issue in management of AID patients. Our results suggested that PCT is an accurate marker for the diagnosis of **bacterial infections and** its peak levels is related to the severity of sepsis. Moreover,delayed PCT clearance may to be a powerful predictor of mortality.

In contrast to previous data, demonstrating no diagnostic value for bacterial infection in AID patients [7, 8], but in line with other reports [9, 10], we detected the utility of PCT for differentiating between bacterial infection and nonbacterial condition. The ambiguous conclusions may be attributable to several factors: First, enrolled difference kind of AID or heterogeneous groups of patients as controls. For example, some studies included in the healthy individuals [9, 10], and others used the full spectrum of AID, or certain AID or disease flares [7, 8, 25, 26] and elevated PCT were found in patients who have certain AID but no evidence of bacterial infection (such as adult-onset Still’s disease [25], Wegener’s [27]). Sencond, the timing of the first PCT measurement varied accounted for by the difficulty in determining the time of onset of sepsis and the time of patient recruitment. In the present study, the peak levels of PCT used may provide better performance based on the potential to detect a late increase of PCT, which is usually seen in the early stages of infections, pretreatment or subacute infections and was also demonstrated by our previous investigation [28]. Third, the definition of infection is a methodological limitation in all similar studies, compared with those using only clinical criteria [7–11], infection was defined microbiologic confirmed may be further improved the diagnostic ability in our setting. All of these may also be responsible for the varied cutoff points of PCT for bacterial infection.

Forthermore, we also report different features of either infection-related PCT response or organ-dysfunction related induction. Also in patients with organ-dysfunction, there may be PCT induction, but this is usually not as high as in bacterial infection at comparable levels of organ dysfunction. This finding indicates that bacterial infection still remains the primarily cause for PCT elevation in critically ill patients. In addition, a significantly positive correlation between severity of sepsis and PCT level will help in high-risk classification.

It is also important to identify markers that provide prognostic information. In the present study, PCT clearance was more useful than absolute level. Similar findings were confirmed by other studies [29–31], however, our study supplemented the work by extended to all patients, not only in septic patients. It is very interesting to note that various conditions other than bacterial infection may induce PCT elevation, for example, severe trauma, prolonged cardiogenic shock, organ dysfunction, which indicate the cytokine-like behavior of PCT during inflammation and infection [32]. As a result, the course of PCT levels over time is a sign of alarm indicating a high risk of organ dysfunction due to systemic inflammation. Therefore, we have reason to believe that failure to decline in the PCT levels, regardless of the induced cause (infection or organ-dysfunction), has been related to higher mortality rates. That is why PCT clearance may influence the prognosis of non-infectious. Published studies in acute cardiac patients or surgery, acute ischemic stroke have come to similar conclusions [33–35].

Several limitations of our study should also be noted. First, we have included a heterogeneous population of AID patients and different cutoff point may be needed in different AID. While this diversity is routine in the ICU. Second, the definition of infection is a methodological limitation and lead to misclassified or bias. Similarly, the fact of antibiotic pretreatment before ICU admission may also influence the identification of the pathogen and misclassify, but we minimized the deviation by using a retrospective diagnosis and excluding the patients who had uncertain diagnosis or no pathogens were identified. Third, a single center study may limit the general application of the results. Further studies are needed to clarify the reliability of PCT kinetics for outcome. Yet, our study is the first to describe a PCT-c as independent determinant of mortality and may play an important role in AID patients management.

## Conclusion

PCT might be as a promising tool for early detection of bacterial infection and risk stratification in critically ill patients with AID. Dynamic change of PCT is associated with prognosis. Additional studies are required to estimate the reliability of threshold and PCT kinetics when used patients management.

## Additional file

Additional file 1: **Additional etiological findings in the patients with or without bacterial infection.** (DOCX 15 kb)

## Abbreviations

AID: Autoimmune diseases
PCT: Procalcitonin
PCT-C: PCT clearance
PCT_peak_: Peak PCT levels within 24 h
SOFA: Sepsis-related Organ Failure Assessment
AUC: The area under the receiver operating characteristic curve
SIRS: Systemic inflammatory response syndrome
SLE: Systemic lupus erythematosus
*DM*: *Dermatomyositis*
*pSS*: *Primary Sjogren’s syndrome*
*RA*: *Rheumatoid arthritis*
*SSc*: *Systemic sclerosis*
*MCTD*: *Mixed connective tissue disease*
*AOSD*: *Adult-onset Still’s disease*
*WG*: *Wegener’s granulomatosis*
*BSI*: *Bloodstream infection*
*CNS*: *Central nervous system*
*CRF*: *Chronic renal failure*
*ARF*: *Acute renal failure*
*DAH*: Diffuse alveolar hemorrhage
*GIB*: *Gastrointestinal bleeding*
*SRC*: *Sclerode rmarenal crisis*
*ILD*: *Interstitial lung disease*
*MRSA*: *Methicillin-resistant Staphylococcus aureus*
*MSSA*: *Methicillin-sensetive Staphylococcus aureus*
*CMV*: *Cytomegalovirus*
