# Supplementary material for: The utility of initial procalcitonin and procalcitonin clearance for prediction of bacterial infection and outcome in critically ill patients with autoimmune diseases: a prospective observational study
Source: BMC Anesthesiol. 2015 Oct 7;15:137. doi: 10.1186/s12871-015-0122-9 (PMC4596456; doi:10.1186/s12871-015-0122-9)
Supplement: Additional file 1: — Additional etiological findings in the patients with or without bacterial infection. (DOCX 15 kb) [file 12871_2015_122_MOESM1_ESM.docx]

**Additional file**  Etiological findings in the patients with or without bacterial infection

| ***Variable*** | | *Patients, No.* |
| --- | --- | --- |
| ***Bacterial pathogens*** | | ***54*** |
| ***Isolated from blood*** | | ***15*** |
| *P. aeruginosa ^a^* | | *2* |
| *A. baumannii ^a^* | | *2* |
| *E. coli ^a^* | | *2* |
| *Klebsiella pneumoniae ^b^* | | *2* |
| *Enterobacter cloacae* | | *1* |
| *Enterococcus faecalis* | | *1* |
| *Enterococcus casseliflavus* | | *1* |
| *MRSA* | | *2* |
| *Serratia marcescens ^a^* | | *1* |
| *Salmonella typhi* | | *1* |
| ***from high quality respiratory tract specimens*** | | ***36*** |
| *A. baumannii* | | *6* |
| *A. baumannii + aspergillus fumigatus* | | *5* |
| *A. baumannii + Zygomycetes* | | *2* |
| *P. aeruginosa* | | *5* |
| *P. aeruginosa + aspergillus fumigatus* | | *1* |
| *Pseudomonas putida* | | *1* |
| *Klebsiella pneumoniae* | | *3* |
| *Klebsiella pneumonia + P jiroveci* | | *2* |
| *Enterobacter cloacae* | | *1* |
| *E. coli* | | *1* |
| *E. coli + P jiroveci* | | *1* |
| *Serratia marcescens* | | *1* |
| *Serratia marcescens + P jiroveci* | | *1* |
| *M.pneumonie* | | *1* |
| *MRSA* | | *2* |
| *MRSA + M.pneumonie* | | *1* |
| *MRSA + aspergillus fumigatus* | | *1* |
| *MRSA + Rhizopus* | | *1* |
| ***Isolated from cerebrospinal fluid*** | | ***3*** |
| *L.monocytogenes* | | *1* |
| *A. baumannii* | | *1* |
| *MSSA* | | *1* |
| ***Nonbacterial pathogens*** | ***29*** | |
| ***Isolated from blood*** | ***4*** | |
| *Candida albicans* | *1* | |
| *Candida glabrata* | *2* | |
| *Candida parapsilosis* | *1* | |
| ***from high quality respiratory tract specimens*** | ***23*** | |
| *P jiroveci* | *10* | |
| *P jiroveci + Aspergillus fumigates* | *2* | |
| *P jiroveci + CMV* | *2* | |
| *Aspergillus fumigates* | *6* | |
| *Zygomycetes* | *1* | |
| *influenza A/H1N1 virus* | *2* | |
| ***Isolated from other specimens*** | ***2*** | |
| *Cerebrospinal fluid of CMV* | *1* | |
| *Systemic infection of EB virus* | *1* | |

*MRSA, methicillin-resistant Staphylococcus aureus ; MSSA, methicillin-sensetive Staphylococcus aureus ;*

*CMV, cytomegalovirus*

^a^ of two cases, one patient suffered bloodstream infection (BSI) originated from respiratory tract;

^b^ all two patients with BSI originated from respiratory tract
